# Cardiac magnetic resonance in histologically proven eosinophilic myocarditis

**DOI:** 10.1186/s12968-023-00979-0

**Published:** 2023-12-18

**Authors:** Pauli Pöyhönen, Johanna Rågback, Mikko I. Mäyränpää, Hanna-Kaisa Nordenswan, Jukka Lehtonen, Chetan Shenoy, Markku Kupari

**Affiliations:** 1 Heart and Lung Center, Helsinki University Hospital and University of Helsinki, Haartmaninkatu 4, 00029 Helsinki, Finland; 2 Radiology, HUS Diagnostic Center, Helsinki University Hospital and University of Helsinki, Helsinki, Finland; 3 Pathology, HUS Diagnostic Center, Helsinki University Hospital and University of Helsinki, Helsinki, Finland; 4 Cardiovascular Division, Department of Medicine, University of Minnesota Medical Center, Minneapolis, MN USA

**Keywords:** Eosinophilic myocarditis, Cardiac magnetic resonance imaging, Endomyocardial biopsy

## Abstract

**Background:**

Eosinophilic myocarditis (EM) is a life-threatening acute heart disease. Cardiac magnetic resonance (CMR) excels in the assessment of myocardial diseases but CMR studies of EM are limited. We aimed to describe CMR findings in histologically proven EM.

**Methods:**

Patients with histologically proven EM seen at an academic center from 2000 through 2020 were identified. Of the 28 patients ascertained, 15 had undergone CMR for diagnosis and constitute our study cohort.

**Results:**

The patients, aged 51 ± 17 years, presented with fever (53%), dyspnea (47%), chest pain (53%), heart block (20%), and blood eosinophilia (60%). On CMR, all 15 patients had myocardial edema with 10 of them (67%) having abnormally high left ventricular (LV) mass as well. LV ejection fraction measured < 50% in 11 patients (73%) and < 30% in 2 (13%), but only 6 (40%) had dilated LV size. Eight patients (53%) had pericardial effusion. LV late gadolinium enhancement (LGE) was found in all but one patient (13/14; 93%). LGE was always multifocal and subendocardial but could involve any myocardial layer. Patients with necrotizing EM by histopathology (n = 6) had higher LGE mass (32.1 ± 16.6% vs 14.5 ± 7.7%, p = 0.050) and more LV segments with LGE (15 ± 2 vs 9 ± 3 out of 17, p = 0.003) than patients (n = 9) without myocyte necrosis. Two patients had LV thrombosis accompanying widespread subendocardial LGE.

**Conclusions:**

In EM, CMR shows myocardial edema and LGE that is typically subendocardial but can involve any myocardial layer. The left ventricle is often non-dilated with moderate-to-severe systolic dysfunction. Pericardial effusion is common. Necrotizing EM presents with extensive myocardial LGE on CMR.

## Background

Eosinophilic myocarditis (EM) is a rare and life-threatening heart disease with multiple etiologies including hypersensitivity reactions, autoimmune or hematologic diseases, cancer, and infections [1]. Its pathogenesis is thought to involve eosinophilic toxic proteins causing acute cardiomyocyte damage, sometimes even substantial necrosis, followed by secondary endocardial thrombosis and, in more chronic cases, late endomyocardial fibrosis [1, 2]. Its clinical manifestations range from fulminant acute myocarditis to chronic restrictive cardiomyopathy [1, 2]. The most severe subtype, acute necrotizing EM, involves considerable mortality and should be identified rapidly to enable life-saving immunosuppressive therapy [1]. Endomyocardial biopsy (EMB) is the only way to definitive diagnosis of EM despite its limitations in cases of patchy myocardial involvement [3].

Cardiac magnetic resonance (CMR) imaging is increasingly used in the diagnostic work-up of myocardial diseases [4]. It can reveal myocardial edema and injury, the hallmarks of acute myocarditis, as well as ventricular dysfunction, pericardial effusion, and intra-cavitary thrombosis, all potentially associated with EM. Due to the rarity of EM, only solitary case-reports exist on CMR findings in histologically proven EM [5–16]. To add insight into the yield of CMR in EM, we identified and systematically re-analyzed all CMR studies from patients with histologically proven EM seen over two decades at our center. Here we describe the salient CMR findings and compare cases with and without histologically proven necrotizing EM.

## Methods

### Study population

**Fig. 1:**
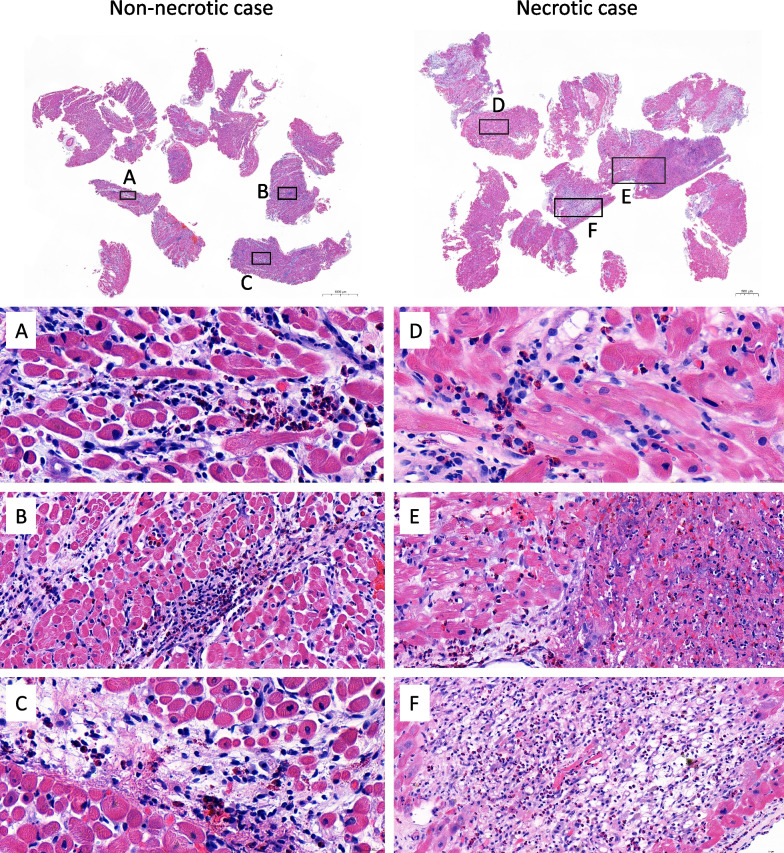
Non-necrotic and necrotic eosinophilic myocarditis. Hematoxylin–eosin staining. Non-necrotic case shows a predominantly eosinophilic infiltrate in all biopsy pieces (**A**–**C**). Eosinophils are partly degranulated, and interstitial oedema is present. Necrotic case shows infiltrating eosinophils in all samples of myocardium (**D**–**F**) with large regions of necrosis (**E**, right side) and granulation tissue (**F**)

The digital pathology reports of Helsinki University Hospital spanning the time from January 2000 to September 2019 (n = 268,341) were screened for potential cases of EM. A total of 497 reports of myocardial histology included findings considered diagnostic of myocarditis or showing inflammatory changes or eosinophilic infiltration. Of these, reports of 34 different cases were identified as likely representing EM. Their histologic specimens were retrieved from Helsinki Biobank and re-analyzed in detail. Ultimately, 28 cases of histologically proven EM were ascertained based on microscopy of myocardial samples taken on EMB (n = 25) or at autopsy (n = 3) showing myocyte damage, interstitial edema, and abundant degranulated and/or intact eosinophils with or without areas of necrosis and/or fibrosis (Fig. 1). Among the 28 histologically proven cases, 27 patients had been hospitalized and one died prior to admission and was diagnosed at autopsy. Fifteen had undergone early CMR for diagnosis. The CMR studies were re-evaluated for the present work, and the hospital charts of all 27 patients were reviewed for clinical data from presentation to end of follow-up in May 2020.

### CMR protocol

The CMR studies were performed with 1.5T or 3T scanners (Avanto, Avanto Fit, Verio and Sonata; Siemens, Erlangen, Germany) at or shortly after presentation using phased-array receiver coils and standard protocols according to contemporary hospital routines [17]. Not all CMR techniques were available in all studies done over the 2-decade study period.

To assess left ventricular (LV) and right ventricular (RV) volumes and ejection fraction (EF), breath-hold cine studies were done using electrocardiographically gated steady‐state free-precession. Cine images were obtained in long-axis (2-, 3- and 4-chamber view) and short-axis planes covering both ventricles (typical slice thickness, 6–8 mm; and interslice gap, 20%). One patient had a temporary pacemaker with active fixation pacing lead during scanning [18].

LGE images were obtained 10 to 15 min after intravenous injection of contrast agent (Dotarem®, Guerbet, Aulnay-sous-Bois, France; 0.15 mmol/kg) using inversion‐recovery gradient echo technique in views identical to the cine studies (inversion time 240–360 ms). First-pass rest perfusion imaging was performed in three short-axis slices (basal, mid, and apical) and an apical four-chamber view. T2-weighted (T2W) fat saturation images/short tau inversion recovery images were regularly obtained. Quantitative myocardial T1 mapping was performed in 1–3 short-axis slices (basal, mid, and apical) using a shortened Modified Look-Locker Inversion-recovery (MOLLI) sequence before and 15 min after gadolinium injection. Hematocrit from the same day was used for extracellular volume fraction measurements. T2 mapping was performed in a breath-hold fashion using three T2-prepared balanced steady state free precession images (T2-preparation times 0 ms, 25 ms, and 55 ms) before gadolinium injection in views identical to T1 mapping.

### CMR analysis

Images were analyzed by a single CMR-trained cardiologist (P.P.) blinded to clinical data and read for consensus in a virtual meeting with another experienced CMR cardiologist (C.S.). Image analysis was performed using QMass MR software® (version 8.1, Medis Medical Imaging Systems, Leiden, the Netherlands). Ventricular volumes, mass and EF were evaluated using standard protocols [19] and compared to UK biobank reference values [20], with abnormal values in women (men) as LV end-diastolic volume > 101 (117) ml/m^2^, LV mass > 55 (72) g/m^2^, LVEF < 50 (47) %, RV end-diastolic volume > 110 (128) ml/m^2^, RVEF < 45 (40) %. Pericardial effusion was defined as > 5 mm pericardial space anterior to the RV wall [21]. Presence of LGE was identified visually. The number of LV segments with LGE was counted according to the AHA 17-segment model [22]. LGE pattern in each segment was classified as subendocardial (including LGE on the RV side of the septum), mid-wall/subepicardial (myocarditis-like LGE) [23], or transmural. Wide-spread subendocardial LGE was defined as ≥ 4 adjacent segments [24]. The extent of LGE as a percentage of LV mass was assessed using the full width at half-maximum method [25]. Multifocality of LGE was defined as more than one discrete lesion within the LV. Insertion site LGE was not counted in multifocality unless clearly continuing into the LV. Finally, in each case, the congruence of the composite CMR findings with the 2018 Lake Louise Criteria for myocarditis [4] was assessed.

### Statistical analysis

Continuous variables are presented as mean ± standard deviation for normally distributed data and median (interquartile range) for skewed data, respectively. Categorical variables are presented as frequency (%). Group comparisons were performed with Student’s t test, Mann–Whitney U test, Chi-square test with continuity correction, or Fisher’s exact test, as appropriate. A *p*-value of < 0.05 was considered statistically significant and all tests were 2-sided. Statistical analysis was performed on R [RStudio, version 4.1.2, The R Foundation; (https://www.r-project.org/)].

## Results

### Characteristics of the study population

Table 1 shows the presenting clinical characteristics and the key laboratory and echocardiographic observations in all 27 patients with EM comparing cases with and without CMR. The study population had a mean age of 52 years. Altogether 14 of the 15 patients undergoing CMR were hospitalized and diagnosed after 2010 while most cases without CMR (8 of 12) presented before 2010 (p = 0.003). The histologic diagnosis of EM was obtained by EMB in all except 2 cases that were detected at autopsy.

**Table 1:** Characteristics of patients with eosinophilic myocarditis with and without cardiac magnetic resonance (CMR) imaging at presentation

	All patients n = 27	CMR+ n = 15	CMR− n = 12	*p*-value
Age, y	52.0 ± 17.2	51.2 ± 16.8	53.1 ± 18.4	0.787
Sex, female, n (%)	11 (41)	6 (40)	5 (42)	1.000
Time from symptom onset to hospital admission, days	3 (1–5)	2 (1–8)	4 (2–5)	0.980
Etiology, n (%)				0.543
Hypersensitivity	10 (37)	4 (27)	6 (50)	
Hypereosinophilic syndrome	7 (26)	4 (27)	3 (25)	
Eosinophilic granulomatosis with polyangitis	3 (11)	2 (13)	1 (8)	
Cancer	1 (4)	0	1 (8)	
Infection^a^	1 (4)	1 (7)	0	
Undefined	5 (19)	4 (27)	1 (8)	
Key laboratory findings on admission				
Peripheral eosinophilia (> 0.44 × 10^9^/L), n (%)	16 (59)	9 (60)	7 (58)	1.000
Elevated cardiac troponins, n (%)^b^	27 (100)	15 (100)	12 (100)	NA
Elevated natriuretic peptides, n (%)^c^	13/16 (81)	9/11 (82)	4/5 (80)	1.000
Electrocardiography				
ST segment elevation or depression, n (%)	12 (44)	7 (47)	5 (42)	0.795
T wave inversions only, n (%)	5 (19)	3 (20)	2 (17)	1.000
Bundle branch block, n (%)	3 (11)	1 (7)	2 (17)	0.569
Atrio-ventricular block, 3rd degree, n (%)	4 (15)	3 (20)	1 (8)	0.605
Echocardiography				
Left ventricular end diastolic diameter, mm, n = 25/14/11	53 ± 10	50 ± 7	58 ± 12	0.088
Interventricular septal thickness, mm, n = 15/10/5	11 (11–15)	12 (11–15)	11 (10–12)	0.286
Left ventricular ejection fraction, %	41.3 ± 17.5	48.8 ± 17.0	31.9 ± 13.5	0.008
<50%, n (%)	15 (56)	5 (33)	10 (83)	0.009
<30%, n (%)	8 (30)	1 (7)	7 (58)	0.008

Data are numbers (%) of cases, means ± standard deviation, or medians (interquartile range)

^a^Non-parasitic infection

^b^Troponin T > 50 ng/L or Troponin I > 125 ng/L

^c^N-terminal brain natriuretic pro-peptide > 300 ng/L or brain natriuretic peptide > 100 ng/L

The presenting signs and symptoms of EM included fever (53% in patients with CMR vs 58% in those without), dyspnea (47% vs 58%), chest pain (53% vs 25%), overt heart failure (33% vs 58%), symptomatic 3rd degree atrio-ventricular block (20% vs 17%) and ventricular tachycardia (7% vs 8%). As shown in Table 1, hypersensitivity reaction was the most common etiology, its causes comprising exposure to drugs including mesalazine (4 cases), amoxicillin, cephalexin, doxycycline, and gefitinib, with in vitro fertilization and alcohol suspected in the remainder of the cases.

On laboratory examinations, blood eosinophil count was abnormally high (> 0.44 × 10^9^/L) in 60% of patients on admission and in 80% during the entire hospitalization. Circulating cardiac troponins were abnormally elevated in all patients, natriuretic peptides being elevated in 82%. A total of 64% of patients had ST segment or T wave abnormalities on 12-lead electrocardiogram. In general, as Table 1 shows, patients with and without CMR for diagnosis had similar characteristics at presentation except that severe LV dysfunction on echocardiography was less prevalent in patients undergoing CMR.

### CMR studies

**Fig. 2:**
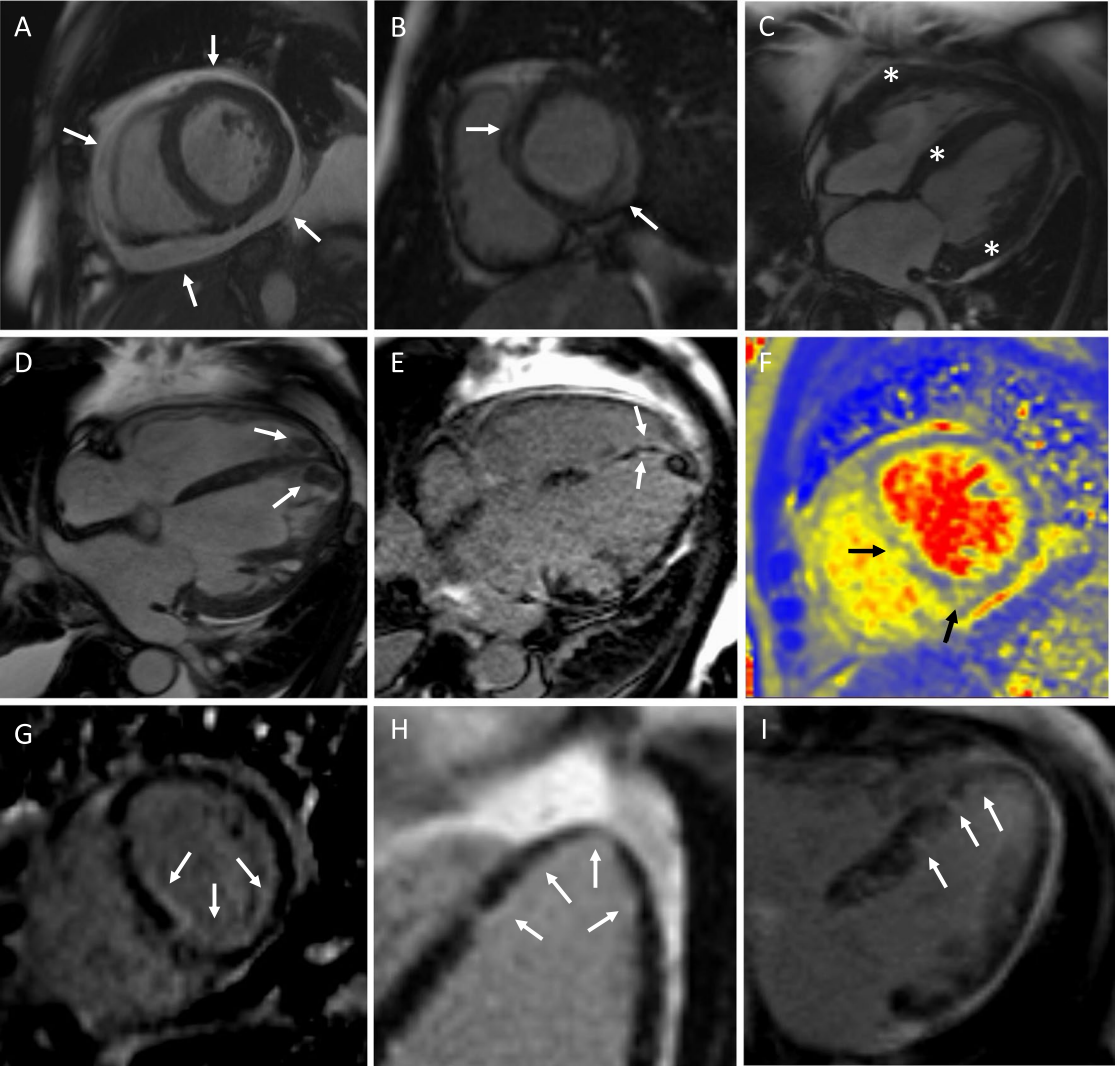
Typical cardiac magnetic resonance (CMR) phenotypes in eosinophilic myocarditis (EM). **A** Marked pericardial effusion on cine images (arrows). **B** Septal and inferolateral mid-wall/subepicardial (myocarditis-like) late gadolinium enhancement (LGE) (arrows). **C** Thickened edematous left ventricular (LV) walls due to severe inflammation on cine images (asterisks). Bi-ventricular apical thrombosis on cine images (**D**) and subendocardial LGE (**E**) (arrows). **F** T2 map showing elevated values (70–80 ms) in septal regions. Patient (**D**–**F**) suffered aborted sudden cardiac death and transient ischemic attack during hospitalization but recovered with mechanical circulatory support, corticosteroids and implantable cardioverter defibrillator. **G**, **H**, **I** Examples of subendocardial LGE in the LV

All studies were performed between March 2004 and August 2019. The median delay from hospital admission to CMR was 1 (range: 1–7) day. The heart rate during cine and LGE imaging averaged 92 ± 20 beats/min and 85 ± 19 beats/min, respectively. Figure 2 is a collage of typical CMR findings in EM, and Table 2 summarizes the CMR data comparing patients with (n = 6) and without (n = 9) overt myocardial necrosis by histopathology.

**Table 2:** Findings by cardiac magnetic resonance in eosinophilic myocarditis with comparison of patients with and without myocardial necrosis on histopathology

	All patients (n = 15)	NEM (n = 6)	Non-NEM (n = 9)	*p*-value
Ventricular volumes, function, wall thickness, and pericardium				
LV end-diastolic volume, ml/m^2^	107 ± 32	121 ± 41	98 ± 21	0.231
LV ejection fraction, %	40 ± 14	33 ± 16	45 ± 12	0.144
LV maximal wall thickness, mm	12.7 (11.1–14.6)	13.6 (13.1–15.1)	11.2 (10.9–12.7)	0.111
LV mass, g/m^2^	72 (63–88)	88 (75–101)	68 (58–72)	0.036
RV end-diastolic volume, ml/m^2^, n = 14/6/8	102 ± 25	112 ± 35	94 ± 12	0.247
RV ejection fraction, % n = 14/6/8	42 ± 14	35 ± 14	49 ± 7.5	0.037
RV free wall maximal thickness, mm	3.9 (3.2–4.2)	4.0 (3.3–4.2)	3.6 (3.3–4.1)	0.679
Pericardial effusion^a^	8 (53)	2 (33)	6 (67)	0.315
Presence of LV thrombus, n	2 (13)	1 (17)	1 (11)	1.000
Presence of RV thrombus, n	1 (7)	1 (17)	0	0.400

	n = 14	n = 6	n = 8	
Late gadolinium enhancement (LGE) imaging^b^				
Presence of LV LGE	13 (93)	6 (100)	7 (88)	1.000
Presence of RV free wall LGE	7 (50)	5 (83)	2 (25)	0.103
LV segments with LGE, n	11 ± 4	15 ± 2	9 ± 3	0.003
With subendocardial involvement, n	5 ± 3	7 ± 1	3 ± 1	< 0.001
With mid-wall/subepicardial involvement, n	3 ± 2	2 ± 2	3 ± 2	0.214
With transmural involvement, n	4 ± 3	6 ± 3	3 ± 2	0.047
With septal involvement, n	5 (4–5)	5 (5–5)	4 (3–5)	0.017
Wide-spread subendocardial LGE, n^c^	7 (54)	5 (83)	2 (29)	0.103
LV LGE mass, %	23 ± 15	32 ± 17	15 ± 7.7	0.0496
Multifocal	13 (100)	6 (100)	7 (100)	NA

Data are numbers (%) of cases, means ± standard deviation, or medians (interquartile range)

*LV* left ventricular, *NEM* necrotizing eosinophilic myocarditis, *RV* right ventricular

^a^> 5 mm effusion anterior to right ventricular wall

^b^Administration of gadolinium was considered contraindicated in 1 patient

^c^≥ 4 adjacent segments

#### Basic CMR characteristics

On cine CMR, 6 of 15 patients (40%) had increased LV diastolic volume and 11 (73%) had LVEF < 50%; severe LV dysfunction (EF < 30%) was found in 2 patients (13%). LV mass exceeded the sex-specific reference range (see Methods) in 10 patients (67%). Altogether, 60% of patients had left ventricular wall thickness of ≥ 12 mm and two patients (13%) right ventricular free wall thickness of > 5 mm. Comparisons between patients with and without myocardial necrosis showed that necrotizing EM was associated with higher LV mass (p = 0.036) and poorer RVEF (p = 0.037).

#### Myocardial injury and edema

**Fig. 3:**
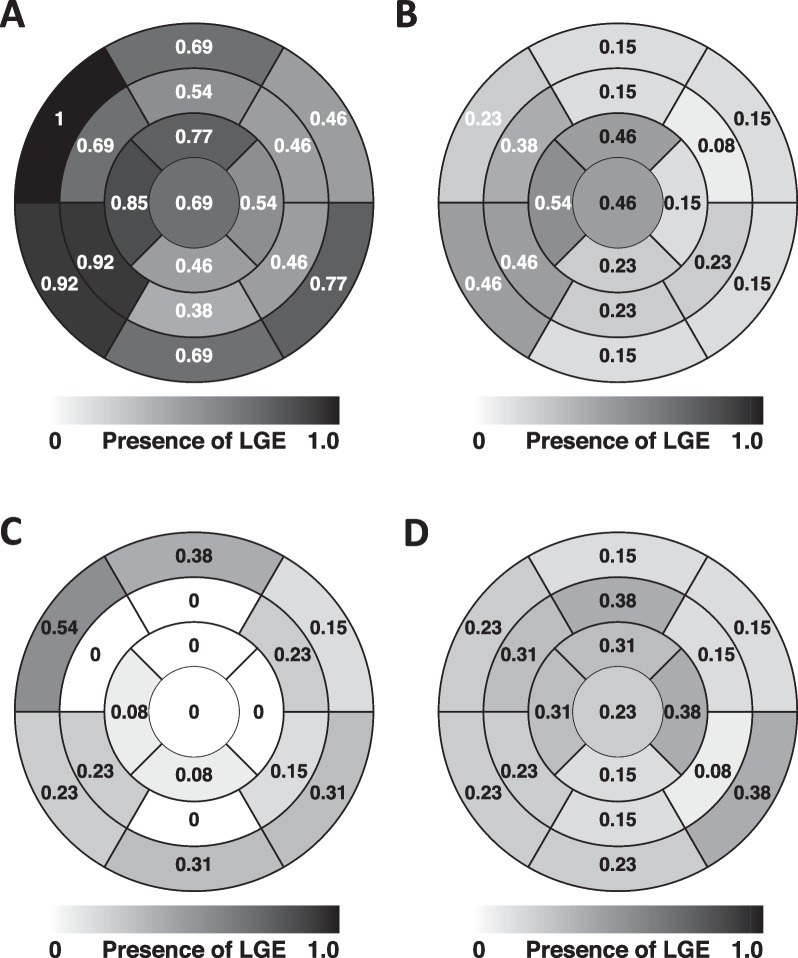
Distribution of left ventricular late gadolinium enhancement (LGE) in eosinophilic myocarditis according to AHA 17-segment model. Numbers inside the segments and the intensity of gray depict the prevalence of LGE (proportion of patients) with any (**A**), subendocardial (**B**), mid-wall/subepicardial (myocarditis-like) (**C**), or transmural (**D**) involvement per segment. *LGE* late gadolinium enhancement

**Fig. 4:**
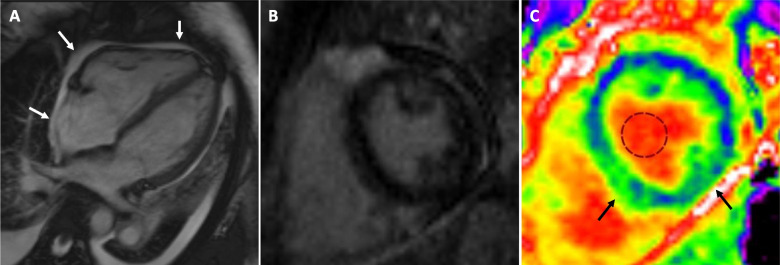
Eosinophilic myocarditis without late gadolinium enhancement. A 27-year old female patient presented with fever and chest pain for 2 days. Cardiac magnetic resonance on 2^nd^ hospital day showed diffuse pericardial effusion (white arrows), normal cavity volumes, and mildly reduced ejection fractions in the left (47%) and right (37%) ventricle (**A**). There was no evident late gadolinium enhancement (**B**) but the image quality was slightly compromised due to the presence of tachycardia (> 100/min) and pericardial effusion. Native T1 values (1150 – 1200 ms diffusely) and extra-cellular volume fractions (30–40%) [black arrows, (**C**)] were elevated indicating myocardial injury

LGE images were available in 14 of 15 patients; T1 and extracellular volume measurements could be done in 11 patients. LGE involvement was invariably diffuse and multifocal with septal, apical, and basal inferolateral segments being most often involved (Fig. 3). The single patient without myocardial LGE had diffusely elevated T1 values (1150–1200 ms) and extracellular volume fractions (30–40%) (Fig. 4). Subendocardial LGE was present in each of the 13 LGE-positive patients of whom 7 (54%) had widespread subendocardial involvement (Fig. 2 E) and 2 had concomitant LV thrombosis (Figs. 2D, E). The mean proportion of enhanced subendocardial segments was 40 ± 17%. Mid-wall/subepicardial and transmural LGE (Fig. 2B) were both found in 85% of patients; the mean proportions of enhanced segments were 33 ± 21% and 27 ± 23%, respectively. As shown in Table 2, patients with necrotizing EM had more LV segments with LGE (15 ± 2 vs 9 ± 3 out of 17, p = 0.003) and higher LGE mass (32.1 ± 16.6% vs 14.5 ± 7.7%, p = 0.050) than patients without myocyte necrosis.

T2-weighted images were available in 14 of 15 patients and T2 maps in 9 patients. These modalities showed myocardial edema in every patient (Fig. 2F). T2 based values were elevated diffusely or in a patchy manner. All patients fulfilled the 2018 Lake Louise Criteria for myocarditis on CMR [4].

### Treatment and outcome in brief

Table 3 summarizes the treatment and outcome data for all EM patients and compares the subgroups with and without CMR for diagnosis. The data highlight the clinical seriousness of EM in showing that as many as 13 out of 27 patients (48%) needed circulatory or mechanical cardiac support. Among the 15 patients undergoing CMR, the 7 needing circulatory support, compared to the rest 8 patients, had lower LV ejection fraction (32 ± 14% vs 48 ± 11%, p = 0.024), higher maximal LV wall thickness [14 (13–19) mm vs 11 (11–13) mm, p = 0.043], lower RV ejection fraction (35 ± 14% vs 49 ± 7.5%, p = 0.037) and trends toward higher LV mass [83 (72–99) g vs 69 (58–73) g, p = 0.072] and higher LGE mass [28 (22–35) % vs 14 (9.1–18) %, p = 0.101]. Altogether 8 patients (30%) either suffered cardiac death (n = 5), underwent transplantation (n = 1), or experienced life-threatening ventricular arrhythmia (n = 2), of which 4 cardiac deaths in patients on inotropic or mechanical support and 1 ventricular fibrillation occurred during hospitalization.

**Table 3:** Treatment and clinical outcome of patients with eosinophilic myocarditis with and without cardiac magnetic resonance (CMR) imaging at presentation

	All n = 27	CMR n = 15	No CMR n = 12	*p*-value
Anti-inflammatory drugs, n (%)				
Corticosteroid	24 (89)	14 (93)	10 (83)	0.569
Azathioprine	3 (11)	2 (13)	1 (8)	1.000
Methotrexate	1 (4)	1 (7)	0	1.000
Cyclosporine	2 (7)	0	2 (17)	0.188
Need for circulatory support	13 (48)	7 (47)	6 (50)	0.863
Inotropes, n (%)	13 (48)	7 (47)	6 (50)	0.863
Mechanical (ECMO), n (%)	5 (19)	3 (20)	2 (17)	1.000
Device therapy, n (%)				
Temporary pacing	3 (11)	3 (20)	0	0.231
Permanent pacemaker (no CRT-D)	2 (7)	1 (7)	1 (8)	1.000
CRT-D	3 (11)	2 (13)	1 (8)	1.000
Outcome				
Follow-up time, y^a^	3.8 (0.7–8.5)	3.6 (1.1–7.0)	4.7 (0.3–11.0)	0.755
Cardiac death	5 (19)	1 (7)	4 (33)	0.139
VF or sustained VT	2 (8)	2 (14)	0	1.000
Transplantation	1 (4)	0	1 (8)	0.444

Data are numbers (%) of cases, means ± standard deviation, or medians (interquartile range)

*CMR* cardiac magnetic resonance, *ECMO* extra-corporeal membrane oxygenation, *CRT-D* cardiac resyncronization therapy and implantable cardioverter-defibrillator, *VF* ventricular fibrillation, *VT* ventricular tachycardia

^a^From symptom onset to event or last out-patient follow-up until May 2020

## Discussion

**Fig. 5:**
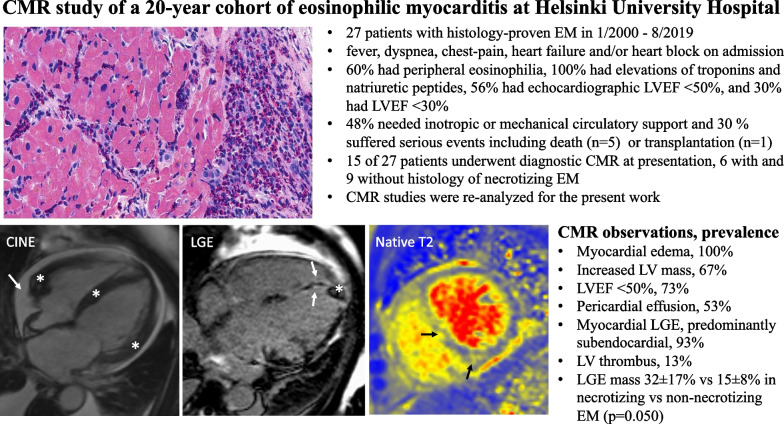
Central Illustration. Cardiac magnetic resonance in eosinophilic myocarditis. Arrows and asterisks highlight the key findings which include pericardial effusion and hypertrophied left ventricular walls in the cine image, subendocardial injury and apical thrombus in the LGE image, and edema in the T2 image. *EM* eosinophilic myocarditis, *LGE* late gadolinium enhancement, *LVEF* left ventricular ejection fraction

We studied CMR findings in histologically proven EM (Fig. 5). The patients were admitted typically with only a few days history of chest pain, dyspnea, and/or arrhythmias, had invariable biomarker signs of myocardial injury and dysfunction, and progressed frequently into circulatory collapse requiring inotropic and mechanical cardiac support but ending fatally in several cases regardless. Their key CMR characteristics were (1) myocardial edema resulting in apparent LV hypertrophy, (2) moderate-to-severe systolic dysfunction of a frequently non-dilated left ventricle, and (3) myocardial LGE that was multifocal and predominantly subendocardial but could involve any myocardial layer. Nearly all patients had LV segments with transmural LGE. Pericardial effusion was common. Of note, patients with necrotizing EM had a clearly higher number of LV segments with subendocardial LGE and more than twice the LGE mass than those without myocardial necrosis on histopathology.

Prior to our work, only solitary case reports existed on CMR imaging in histology-proven EM [5–16]. Recently, Antonopoulos et al. [24] reported CMR findings in a large retrospective cohort of patients with peripheral eosinophilia, but the histologic equivalents of imaging characteristics could not be confirmed as myocardial biopsies were not done. The earlier case studies [5–16] showed CMR abnormalities varying by the etiology of EM. Marked subendocardial LGE, with or without intracavitary thrombus, appeared typical for cases of chronic eosinophilia [8–10, 15], while more acute cases were often associated with myocardial edema, prolonged values of T1 and T2, and patchy LGE that was predominantly subepicardial [5, 6, 11] but could involve LV subendocardium as well [12]. These observations fit well inside the spectrum of our data. Curiously, and in striking discord with our work, cases of acute necrotizing EM free of any CMR abnormality, save for small pericardial effusion, have also been reported [13, 14]. None of our patients had normal or near normal CMR.

Acute necrotizing EM shares many clinical characteristics and some histologic features with giant cell myocarditis (GCM) [26, 27], another possible cause of fulminant heart failure. Both can present with a short history of arrhythmias, heart block, and heart failure and may rapidly evolve into circulatory collapse needing inotropic or mechanical cardiac support. Lack of peripheral eosinophilia in a significant proportion of EM [1], in 40% of our patients, complicates their differentiation further as does the myocardial histology of GCM often including increased eosinophils along with giant cells, myocyte necrosis and fibrosis [27, 28]. The present observations and our recent findings in GCM [29] show that there are no CMR characteristics truly distinctive of either condition, although subendocardial LGE in the apical LV segments may be more common in EM while localized areas of myocardial thinning, septal in particular, favor GCM [29]. In clinical practice, reliable differentiation of EM from other causes of fulminant or non-fulminant myocarditis requires myocardial biopsy and histology. In the presence of visible endocardial thrombus, the risks and benefits of EMB must be carefully weighed, though. In our study population, endocardial thrombi were rare observations in either ventricle (Table 2).

There was a numerical, though statistically non-significant, trend of more chest pain (53% vs 25%) and less overt heart failure (33% vs 58%) in EM patients with CMR compared to those without CMR. Increased availability of CMR during the study (93% of CMRs were done after 2010) and the move of the initial diagnostic modality from EMB to CMR may have changed the observed spectrum of clinical manifestations, some patients with chest pain eluding diagnosis before the CMR era.

### Limitations

The main strength of our study is its design—a systematic analysis of CMRs in a 20-year cohort of patients cared in an academic hospital due to histologically proven EM. Although our findings likely are less influenced by selection bias than solitary case reports, comparison of cases with and without CMR (Table 1) suggest that patients with poorest LV function and most severe cardiovascular compromise could not undergo CMR imaging. Thus, some of the sickest patients with EM may have been missed for this reason. The median time from symptom onset to hospitalization was only few days in this series. More chronic forms of EM may not fulfill all of the Lake Louise Criteria for myocarditis. Our patients did not receive anti-interleukin-5 therapies. Other limitations of our work include its retrospective design, the small number of patients, lack of follow-up CMR examinations, and lack of control groups of other types of fulminant or non-fulminant myocarditis. LGE with long inversion time, optimized for the detection of small intra-cavitary thrombi, was not used in this study.

## Conclusions

The take-home conclusions from our work are, first, that acute EM is a genuinely life-threatening cardiac condition that is frequently caused by hypersensitivity to drugs and can present without peripheral eosinophilia and deteriorate within a few days into circulatory failure involving in-hospital mortality. Second, CMR imaging in EM, if feasible, effectively exposes the general signs of myocarditis including myocardial edema and injury with ventricular dysfunction and pericardial effusion. Further, the extent of myocardial LGE correlates with myocardial histology with extensive LGE mass suggesting necrotizing EM. However, no findings on CMR are specific to EM and its diagnosis and distinction from other types of myocarditis requires myocardial biopsy and histopathology.

## Data Availability

The data underlying this article cannot be shared publicly due to restrictions by the patient consent.

## Abbreviations

CMR: Cardiac magnetic resonance
CRT-D: Cardiac resynchronization therapy and implantable cardioverter-defibrillator
EF: Ejection fraction
EM: Eosinophilic myocarditis
EMB: Endomyocardial biopsy
GCM: Giant cell myocarditis
LGE: Late gadolinium enhancement
LV: Left ventricular
RV: Right ventricular
